# Draft Genome Sequences of the *Mycobacterium tuberculosis* Clinical Strains A2 and A4, Isolated from a Relapse Patient in Taiwan

**DOI:** 10.1128/genomeA.00672-14

**Published:** 2014-09-04

**Authors:** Yu-Chieh Liao, Yih-Yuan Chen, Hsin-Hung Lin, Jia-Ru Chang, Ih-Jen Su, Tsi-Shu Huang, Horng-Yunn Dou

**Affiliations:** a National Institute of Infectious Diseases and Vaccinology, National Health Research Institutes, Zhunan, Miaoli, Taiwan; b Division of Biostatistics and Bioinformatics, Institute of Population Health Sciences, National Health Research Institutes, Zhunan, Miaoli, Taiwan; c Department of Microbiology, Kaohsiung Veterans General Hospital, Kaohsiung, Taiwan

## Abstract

The recurrence rate of *Mycobacterium tuberculosis* in Taiwan is 3%. Here, we present the draft genome sequences of *M. tuberculosis* strains A2 and A4 from a relapse patient. The draft genome sequences comprise 4,443,031 bp and 4,487,096 bp, revealing 4,220 and 4,143 coding sequences for A2 and A4, respectively, as well as 49 tRNA genes for the both isolates.

## GENOME ANNOUNCEMENT

Resulting in approximately 9.4 million new cases and 1.7 million deaths each year, tuberculosis (TB) remains a global health concern (1). Although anti-TB drugs are effective at treating patients with good compliance, the recurrence rate in Taiwan is still around 3% (2). Several host factors, such as HIV infections and ethnicity, predispose cured patients to relapse (3). In addition, bacterial factors may also play a role. Therefore, we aim to compare the genomes of the relapsed isolates with those with the same genotype. We hope to identify the virulence gene that contributes to relapse. Relapse cases were defined as patients who were cured by a full course of anti-TB drug treatment and then had a recurrence of *Mycobacterium tuberculosis* infection during a follow-up period, with bacteriological confirmation. The fine changes in the genomic sequence may help *M. tuberculosis* evade the immune system. Therefore, an analysis of different stages of mycobacterial genome variation will also help pinpoint how isolated strains evade host immunity.

In this study, we present the completed genome sequence of the clinical isolates *M. tuberculosis* A2 and A4, isolated at Kaohsiung Veterans General Hospital, Taiwan, from a human sputum sample of a relapsed patient, which was confirmed at different stages to be tuberculosis. Strains A2 and A4 were also analyzed by spoligotyping (777777777720771) and mycobacterial interspersed repetitive unit–variable-number tandem-repeat (MIRU-VNTR) typing (314222325163223234233262), identifying them as belonging to the Haarlem lineage and confirming that the two strains indeed belong to the same clone.

This study was approved by the Human Ethics Committee of the National Health Research Institutes, Taiwan (code EC1010804-E). Because of the retrospective nature and deidentification of personal information of the subjects, the requirement of obtaining informed consent was waived by our institutional review board.

Genomic DNA of both isolates was extracted from cultured cells as described previously (4) and sequenced by the MiSeq platform (Illumina, USA) to generate 8,282,272 and 11,387,732 paired-end reads for A2 and A4, respectively. The sequencing reads (length, 251 bp) were trimmed and discarded by limiting the quality score at 0.05 and permitting two ambiguous nucleotides at most in the minimum length of 50 bp. The filtered reads were assembled into contigs using *de novo* assemblers, including ABySS 1.3.4 (5), Edena version 3.130110 (6), SPAdes 2.5.0 (7), and Velvet 1.2.09 (8). The contigs from the assemblies were then integrated by CISA (9), resulting in final sets of 190 and 166 contigs for A2 and A4, respectively. Please note that because an extra-large genome size (>7 Mb) was produced by SPAdes in assembling A2, we excluded the assembly of SPAdes from the contig integration of A2 using CISA. The draft genome assemblies of 4,443,031 bp and 4,487,096 bp were annotated by Prokka (10) to contain 4,220 and 4,143 coding sequences for A2 and A4, respectively, as well as 49 tRNA genes for both isolates.

### Nucleotide sequence accession numbers.

The whole-genome shotgun projects have been deposited at GenBank under accession numbers JNGF00000000 and JNNW00000000 for A2 and A4, respectively. The versions described in this paper are in the first versions, JNGF01000000 and JNNW01000000. The BioProject ID is PRJNA248335.
